# A retrospective analysis of the incidence and risk factors of perioperative urinary tract infections after total hysterectomy

**DOI:** 10.1186/s12905-024-03153-5

**Published:** 2024-05-29

**Authors:** Xianghua Cao, Yunyun Tu, Xinyao Zheng, Guizhen Xu, Qiting Wen, Pengfei Li, Chuan Chen, Qinfeng Yang, Jian Wang, Xueping Li, Fang Yu

**Affiliations:** 1 Department of Anesthesiology, Dongguan Tungwah Hospital, Dongguan, China; 2 Department of Anesthesia, Longyan First Affiliated Hospital of Fujian Medical University, Longyan, Fujian 364000 China; 3 Department of Dermatology, Nanfang Hospital, Southern Medical University, Guangzhou, Guangdong 510515 China; 4 Department of Obstetrics and Gynecology, Core Facility Center, Division of Life Sciences and Medicine, The First Affiliated Hospital of USTC, University of Science and Technology of China, Hefei, Anhui 230001 China; 5 Division of Orthopaedic Surgery, Department of Orthopaedics, Nanfang Hospital, Southern Medical University, Guangzhou, Guangdong 510515 China; 6 Division of Orthopaedic Surgery, People’s Hospital of Ganzhou, No. 17 Hongqi Avenue, Zhanggong District, Ganzhou, 341000 China

**Keywords:** Perioperative urinary tract infections, Total hysterectomy, Risk factors, Incidence, Nationwide inpatient sample

## Abstract

**Introduction:**

Perioperative urinary tract infections (PUTIs) are common in the United States and are a significant contributor to high healthcare costs. There is a lack of large studies on the risk factors for PUTIs after total hysterectomy (TH).

**Methods:**

We conducted a retrospective study using a national inpatient sample (NIS) of 445,380 patients from 2010 to 2019 to analyze the risk factors and annual incidence of PUTIs associated with TH perioperatively.

**Results:**

PUTIs were found in 9087 patients overall, showing a 2.0% incidence. There were substantial differences in the incidence of PUTIs based on age group (*P* < 0.001). Between the two groups, there was consistently a significant difference in the type of insurance, hospital location, hospital bed size, and hospital type (*P* < 0.001). Patients with PUTIs exhibited a significantly higher number of comorbidities (*P* < 0.001). Unsurprisingly, patients with PUTIs had a longer median length of stay (5 days vs. 2 days; *P* < 0.001) and a higher in-hospital death rate (from 0.1 to 1.1%; *P* < 0.001). Thus, the overall hospitalization expenditures increased by $27,500 in the median ($60,426 vs. $32,926, *P* < 0.001) as PUTIs increased medical costs. Elective hospitalizations are less common in patients with PUTIs (66.8% vs. 87.6%; *P* < 0.001). According to multivariate logistic regression study, the following were risk variables for PUTIs following TH: over 45 years old; number of comorbidities (≥ 1); bed size of hospital (medium, large); teaching hospital; region of hospital(south, west); preoperative comorbidities (alcohol abuse, deficiency anemia, chronic blood loss anemia, congestive heart failure, diabetes, drug abuse, hypertension, hypothyroidism, lymphoma, fluid and electrolyte disorders, metastatic cancer, other neurological disorders, paralysis, peripheral vascular disorders, psychoses, pulmonary circulation disorders, renal failure, solid tumor without metastasis, valvular disease, weight loss); and complications (sepsis, acute myocardial infarction, deep vein thrombosis, gastrointestinal hemorrhage, pneumonia, stroke, wound infection, wound rupture, hemorrhage, pulmonary embolism, blood transfusion, postoperative delirium).

**Conclusions:**

The findings suggest that identifying these risk factors can lead to improved preventive strategies and management of PUTIs in TH patients. Counseling should be done prior to surgery to reduce the incidence of PUTIs.

**The manuscript adds to current knowledge:**

In medical practice, the identification of risk factors can lead to improved patient prevention and treatment strategies. We conducted a retrospective study using a national inpatient sample (NIS) of 445,380 patients from 2010 to 2019 to analyze the risk factors and annual incidence of PUTIs associated with TH perioperatively. PUTIs were found in 9087 patients overall, showing a 2.0% incidence. We found that noted increased length of hospital stay, medical cost, number of pre-existing comorbidities, size of the hospital, teaching hospitals, and region to also a play a role in the risk of UTI’s.

**Clinical topics:**

Urogynecology

**Supplementary Information:**

The online version contains supplementary material available at 10.1186/s12905-024-03153-5.

## Introduction

Approximately 600,000 women in the United States undergo total hysterectomy (TH) each year, with about 10% opting for subtotal (cervix-preserving) procedures [1–3]. A majority of TH are conducted to address non-cancerous conditions like leiomyomas, abnormal uterine bleeding, and endometriosis, whereas only around 10% are carried out as cancer treatment [3, 4]. In 17–23% of cases [2], there are intraoperative and postoperative problems such as bleeding, infection, and visceral injury.

In the United States, urinary tract infections (UTIs) are prevalent and a major cause of high healthcare expenses; in 2010, direct costs from UTIs exceeded $5 billion [5, 6]. Women experience higher rates of UTIs than men do due to anatomical and physiological variations [5, 7, 8]. It has been suggested that the rate of postoperative UTIs is a good measure of surgical quality [9]. In 2012, the Centers for Medicare & Medicaid Services and the Joint Commission on Accreditation of Healthcare Organizations required all Medicare providers to report surgical site infection rates, including UTIs, in a public registry [10]. There is a lack of published research on identified risk factors that could be utilized to calculate the incidence of perioperative urinary tract infections (PUTIs) after TH [10, 11].

This study’s goals were to find out what percentage of women have PUTIs after TH surgery and associated risk factors.

## Materials and methods

### Data source

The national inpatient sample (NIS) database, the largest all-payer database of inpatient admissions in the US, served as the study’s data source. It is a component of the Agency for Healthcare Research and Quality’s Healthcare Cost and Utilization Project. Annually, the NIS obtains a stratified sample of 20% of hospital stays from over 1000 US hospitals [12, 13]. The International Classification of Diseases (ICD) 9th and 10th editions’ major surgical codes are used to identify patients whose primary procedure is a TH (ICD-9 and ICD-10). Information up until the end of 2015 is coded using ICD-9, while information beyond that through the end of 2016 is coded using ICD-10, in accordance with US coding standards. The data that are currently available include demographics, diagnosis and insurance information, hospital details, length of stay (LOS), total charges, and status of discharge (Table 1). Data that has been de-identified is released to the public. Therefore, this study was deemed exempt by the institutional review board.

**Table 1:** Variables used in binary logistic regression analysis

Variables Categories	Specific Variables
**Patient demographics**	Age (18-44years, 45-64years, 65-74years and ≥ 74 years), race (White, Black, Hispanic, Asian or Pacific Islander, Native American and Other)
**Hospital characteristics**	Type of admission (non-elective, elective), bed size of hospital (small, medium, large), teaching status of hospital (nonteaching, teaching), location of hospital (rural, urban), type of insurance (Medicare, Medicaid, private insurance, self-pay, no charge, other), location of the hospital (northeast, Midwest or north central, south, west)
**Comorbidities**	AIDS, alcohol abuse, deficiency anemia, rheumatoid diseases, chronic blood loss anemia, congestive heart failure, chronic pulmonary disease, coagulopathy, depression, diabetes (uncomplicated), diabetes (with chronic complications), drug abuse, hypertension, hypothyroidism, liver disease, lymphoma, fluid and electrolyte disorders, metastatic cancer, neurological disorders, obesity, paralysis, peripheral vascular disorders, psychoses, pulmonary circulation disorders, renal failure, solid tumor without metastasis, peptic ulcer disease, valvular disease and weight loss

AIDS: Acquired immunodeficiency syndrome

### Study population

**Fig. 1:**
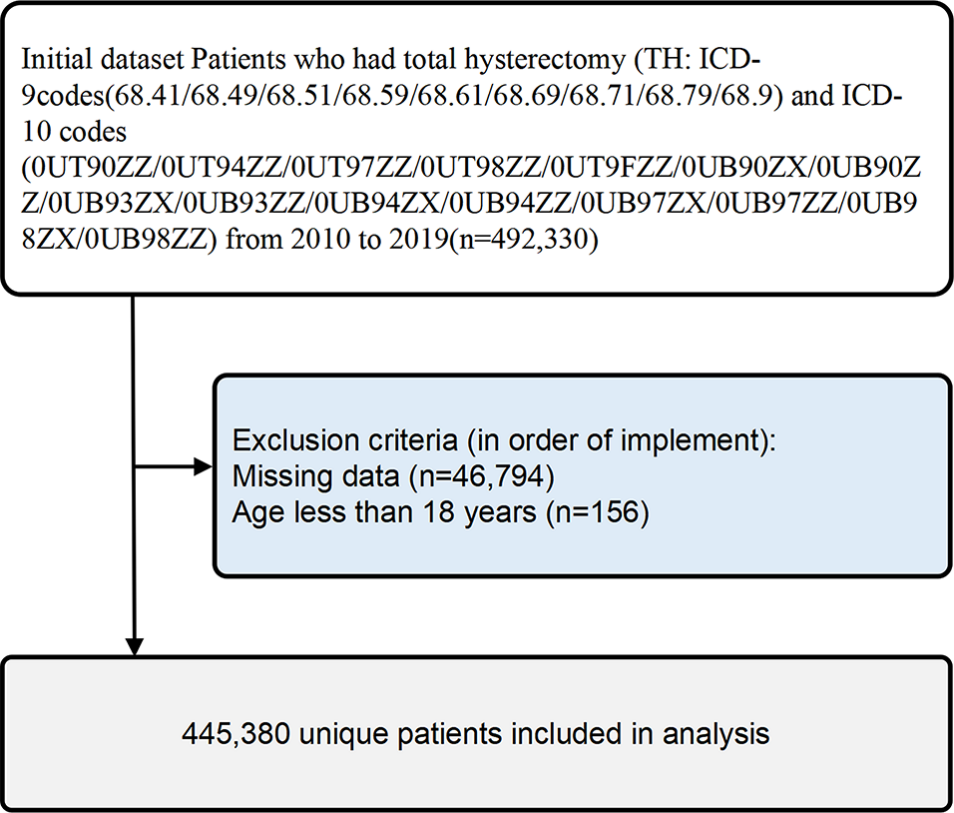
Flow and inclusion/exclusion of all patients had TH

Individuals that underwent TH procedures as indicated by ICD-9 codes (68.41/68.49/68.51/68.59/68.61/68.69/68.71/68.79/68.9) and ICD-10 codes (0UT90ZZ/0UT94ZZ/0UT97ZZ/0UT98ZZ/0UT9FZZ/0UB90ZX/0UB90ZZ/0UB93ZX/0UB93ZZ/0UB94ZX/0UB94ZZ/0UB97ZX/0UB97ZZ/0UB98ZX/0UB98ZZ) from 2010 to 2019 (*n* = 492,330). Patients with missing data (*n* = 46,794) or fewer than 18 years old (*n* = 156) were not included in this study. Most of the missing values in this study were based on patient characteristics in the NIS database, and no data were missing for comorbidities and complications, and any missing data were excluded in this study (Fig. 1). Ultimately, 445,380 individual patients are included in the analysis. Recruits were split into two groups according to whether or not they had PUTIs.

### Outcomes-the postoperative complications

ICD-9-CM and ICD-10-CM diagnostic codes were used to find postoperative complications that could be independently related to PUTIs, such as surgical and medical perioperative issues before discharge. Sepsis, acute myocardial infarction, deep vein thrombosis, gastrointestinal hemorrhage, pneumonia, and stroke were all considered perioperative medical problems. Wound infection, wound rupture, bleeding, pulmonary embolism, blood transfusion, and postoperative delirium were among the perioperative medical problems.

### Covariates

Age and race of the patient, hospital features (such as kind of hospital admission, bed capacity, teaching/non-teaching status, location, insurance type, and hospital location), and comorbidities were among the factors. Classification of non-elective/elective admissions, which can potentially separate emergency from elective care. Elective hospitalization refers to planned admissions for medical procedures or treatments that are scheduled in advance and are not considered urgent or emergent. Non-elective hospitalization, also known as emergency or unplanned hospitalization, involves admissions for acute medical conditions or urgent healthcare needs that require immediate attention [14, 15]. The Charlson Comorbidity Score was used to find 29 comorbidities that existed before surgery. These were then put into groups using ICD-9-CM and ICD-10-CM diagnosis codes (Table 1).

### Data analysis

IBM SPSS Statistics 25.0 was used for all data analysis. The demographic and baseline attributes of the subjects were presented as means and standard deviation, or, when applicable, as median and interquartile range for continuous variables, and frequencies and percentages for categorical data. The Chi-square test was used for nominal and ordinal data, and the Student’s t test was used to compare continuous variables. The study used multivariate logistic regression to survey the national inpatient sample (NIS) database of women and find these risk factors (patient demographics, hospital features, preoperative comorbidities, and surgical and medical perioperative problems). The odds ratios (ORs) of baseline parameters and any surgical and medical problems compared between the two groups were computed using binary logistic regression. *P* < 0.001 was utilized to establish the statistical significance of the alpha level because previous NIS research had used high sample numbers [16]. Images were drawn using Free Statistics software version 1.9. and pptx. of Word Processing System (WPS) Office (Kingsoft) tools.

## Results

### Incidence of PUTIs in patients undergoing TH

**Fig. 2:**
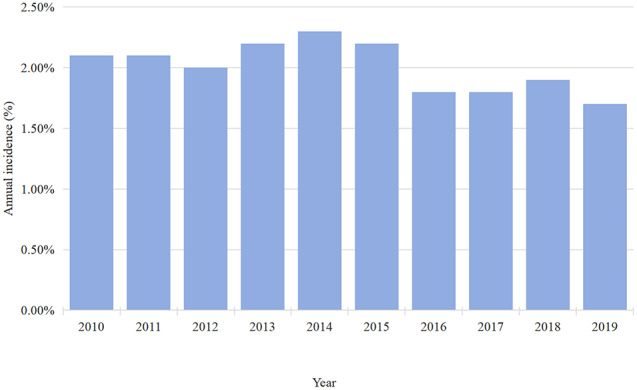
Annual incidence of PUTIs after TH

Between 2010 and 2019, data on 455,380 TH cases were available in the NIS database. In total, 9087 patients had PUTIs, indicating a 2.0% incidence (Table 2). According to our research, the 2.1% incidence of PUTIs persisted in 2010–2011. According to the study, there was an annual decline in the incidence of PUTIs from 2015 (2.2%) to 2019 (1.7%), but an overall increase in the incidence from 2012 (2.0%) to 2014 (2.3%) (Fig. 2).

**Table 2:** Patient characteristics and outcomes after TH (2010–2019)

Characteristics	PUTIs	No PUTIs	*P*
**Total (n = count)**	9087	436,293	
**Total incidence (%)**	2.0		
**Age (median, years)**	55 (44–68)	47(41–56)	< 0.001
**Age group n (%)**			
18–44	2393(26.3)	175,151(40.1)	< 0.001
45–64	3749(41.3)	200,584(46.0)	
65–74	1725(19.0)	42,028(9.6)	
≥ 75	1220(13.4)	18,530(4.2)	
**Race n (%)**			
White	5524(60.8)	258,776(59.3)	0.02
Black	1691(18.6)	85,128(19.5)	
Hispanic	1212(13.3)	60,040(13.8)	
Asian or Pacific Islander	298(3.3)	14,787(3.4)	
Native American	61(0.7)	2263(0.5)	
Other	301(3.3)	15,299(3.5)	
**Number of Comorbidity n(%)**			
0	1457(16.0)	156,819(35.9)	< 0.001
1	1831(20.1)	125,243(28.7)	
2	1744(19.2)	78,118(17.9)	
≥ 3	4055(44.6)	76,113(17.4)	
**LOS (median, d)**	5 (3–10)	2 (1–3)	< 0.001
**TOTCHG (median, $)**	60,426 (33,653 − 113,929)	32,926 (21,063 − 52,122)	< 0.001
**Type of insure n(%)**			
Medicare	3167(34.9)	69,834(16)	< 0.001
Medicaid	1518(16.7)	67,936(15.6)	
Private insurance	3592(39.5)	263,847(60.5)	
Self-pay	475(5.2)	15,584(3.6)	
No charge	80(0.9)	3066(0.7)	
Other	255(2.8)	16,026(3.7)	
**Bed size of hospital n(%)**			
Small	1001(11.0)	62,122(14.2)	< 0.001
Medium	2397(26.4)	120,497(27.6)	
Large	5689(62.6)	253,674(58.1)	
**Elective admission n(%)**	6070(66.8)	382,398(87.6)	< 0.001
**Type of hospital, teaching n( %)**	6302(69.4)	263,634(60.4)	< 0.001
**Location of hospital, urban n( %)**	8475(93.3)	394,723(90.5)	< 0.001
**Region of hospital n(%)**			
Northeast	1638(18.0)	79,483(18.2)	0.081
Midwest or North Central	1718(18.9)	80,731(18.5)	
South	3830(42.1)	180,308(41.3)	
West	1901(20.9)	95,771(22)	
**Died n(%)**	104(1.1)	474(0.10)	< 0.001

LOS: Length of stay, TOTCHE: Total charge, TH: Total hysterectomy, PUTIs: Perioperative urinary tract infections, No PUTIs: No perioperative urinary tract infections

### Patient demographics and hospital characteristics between the two groups

There was a significant difference in the incidence of PUTIs by age group (Table 2), with a larger proportion of people over 65 years old reporting PUTIs than people under 65 years old (*P* < 0.001). The kind of insurance, hospital bed size, hospital location, and hospital type continuously differed significantly between the two groups (Table 2). However, there were no statistically significant differences in terms of hospital region or race (Table 2). As was previously mentioned, patients with PUTIs had a noticeably higher number of comorbidities (*P* < 0.001). The fact that PUTIs caused an increase in in-hospital mortality from 0.1 to 1.1% (*P* < 0.001) (Table 2). Table 2 shows that the median length of stay (LOS) for patients with PUTIs was greater than that of patients without PUTIs (5 days vs. 2 days; *P* < 0.001). According to Table 2, there was a $27,500 increase in the median hospitalization expenditures for PUTIs ($60,426 vs. $32,926, *P* < 0.001). Furthermore, Table 2 shows that patients with PUTIs have a decreased likelihood of elective hospital admissions (66.8% vs. 87.6%; *P* < 0.001).

### Risk factors between PUTIs and patient demographics

**Fig. 3:**
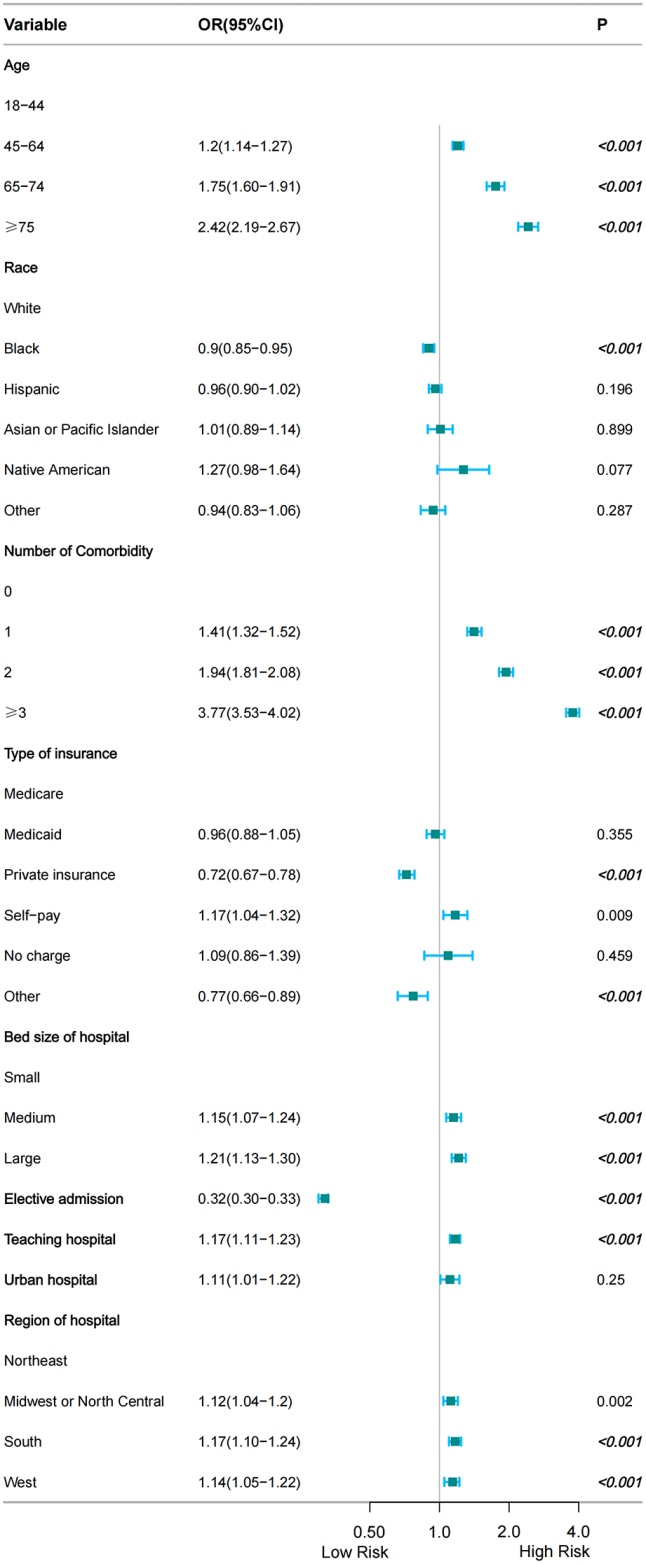
Risk factors associated with PUTIs after TH

The following indicators were found by using multivariate logistic regression analysis to investigate risk factors associated with PUTIs (Fig. 3, Table S1): advanced age; number of comorbidity = 1, number of comorbidity = 2, number of comorbidity ≥ 3; medium bed size of hospital, large bed size of hospital; teaching hospital; south of hospital, west of hospital (Fig. 3, Table S1).

Interestingly, three protective factors were found to be associated with PUTIs, elective admission, black population and private insurance (Fig. 3, Table S1).

### Risk factors between PUTIs and other preoperative comorbidities

**Fig. 4:**
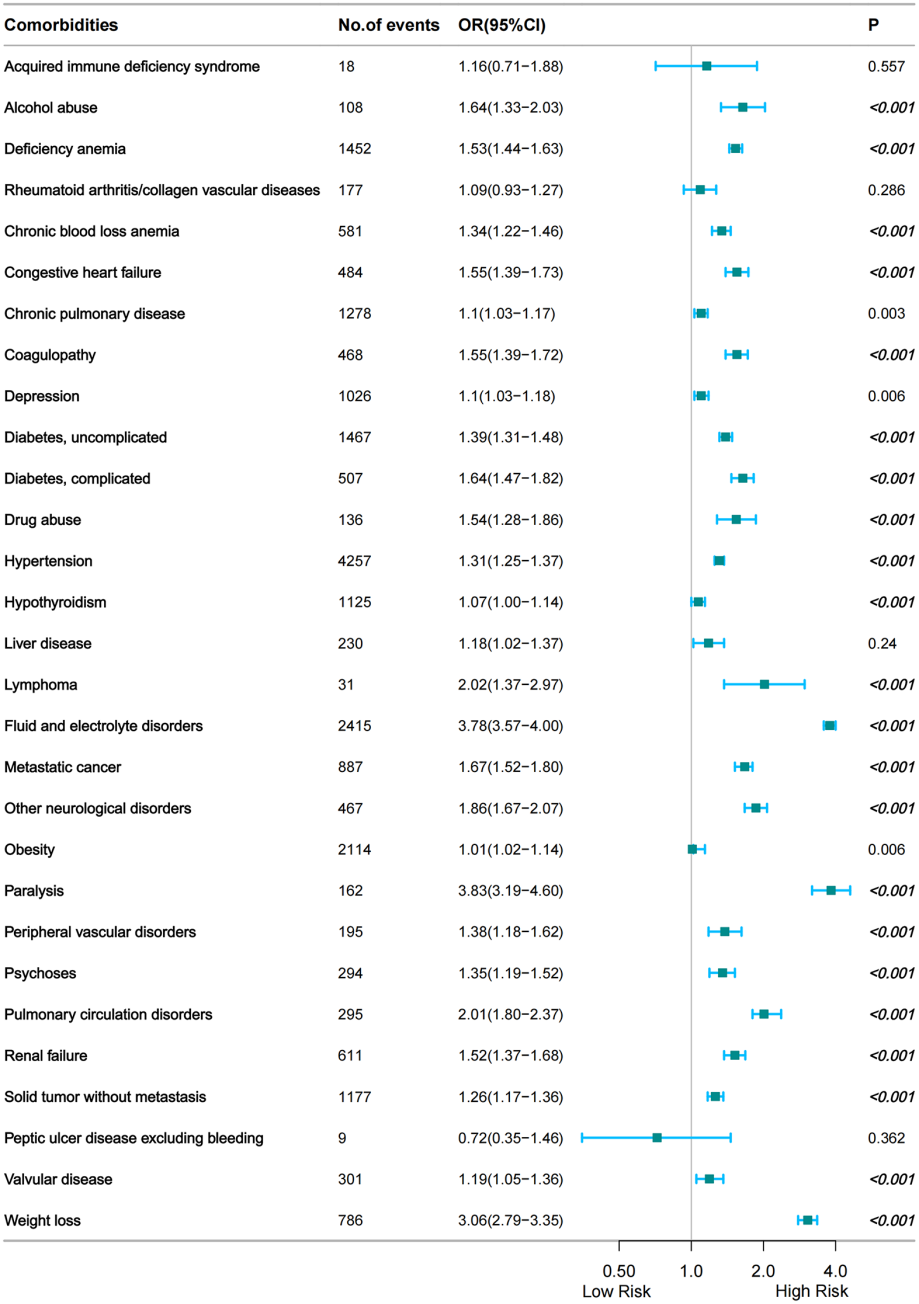
Comorbidities associated with PUTIs following TH

The results of multivariate logistic regression analysis were as follows: alcohol abuse, deficiency anemia, chronic blood loss anemia, congestive heart failure, coagulopathy, diabetes, drug abuse, hypertension, lymphoma, fluid and electrolyte disorders, metastatic cancer, other neurological disorders, paralysis, peripheral vascular disorders, psychoses, pulmonary circulation disorders, renal failure, solid tumor without metastasis, valvular disease weight loss (Fig. 4, Table S2).

### Risk factors between PUTIs and other postoperative complications

**Fig. 5:**
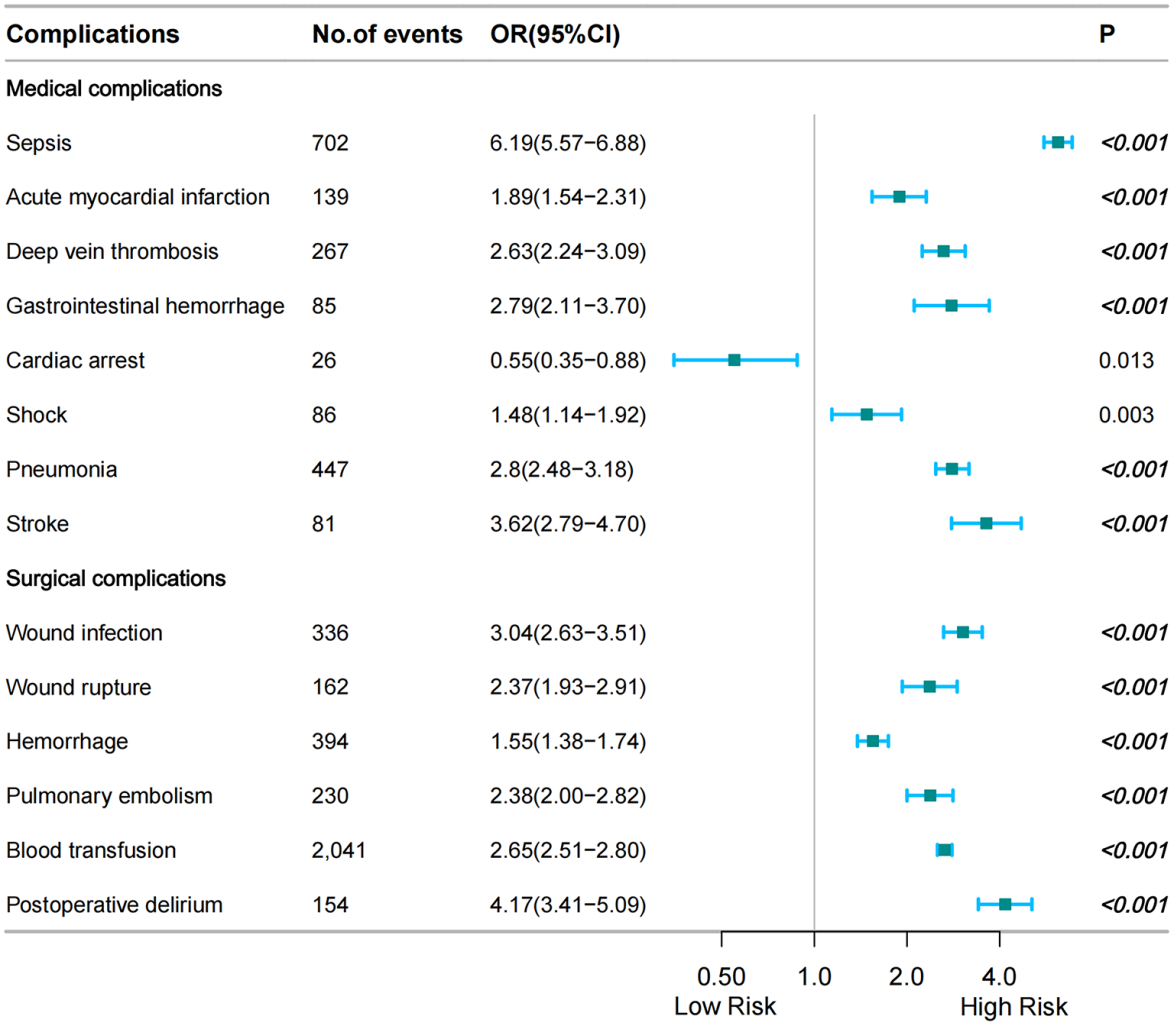
Complications associated with PUTIs following TH

Medical complications of multivariate logistic regression analysis were as follows: sepsis, acute myocardial infarction, deep vein thrombosis, gastrointestinal hemorrhage, pneumonia, stroke (Fig. 5, Table S3); However, in multivariate regression analysis, cardiac arrest seemed to be associated with perioperative urinary tract infection (OR = 0.55, 95%CI = 0.35–0.88, *P* = 0.013), but it was not statistically significant(Fig. 5, Table S3).

Surgical complications of multivariate logistic regression analysis were as follows: wound infection, wound rupture, hemorrhage, pulmonary embolism, blood transfusion, postoperative delirium (Fig. 5, Table S3).

## Discussion

We found the overall occurrence of PUTIs after TH to be 2% (1.7 − 2.3%) between 2010 and 2019 using the NIS database. We identified the following risk factors were associated with the occurrence of PUTIs: age, number of pre-existing comorbidities, size of the hospital, teaching hospitals, and complications. An extensive health economics analysis of PUTIs following TH is provided in the present study. As far as the authors know, this is the first investigation into the prevalence and possible risk factors of PUTIs following a TH. The findings suggest that identifying these risk factors can lead to improved preventive strategies and management of PUTIs in TH patients.

The study found that 2.0% of patients had PUTIs following a TH. Previous research has shown that the incidence of UTIs was less than 5% when the majority of included individuals underwent laparoscopic or abdominal TH [5, 10, 17, 18]. The incidence of PUTIs stayed at 2.1% throughout the 2010–2011 period, then typically grew from 2012 (2.0%) to 2014 (2.3%) (Fig. 2), and then dropped yearly from 2015 (2.2%) to 2019 (1.7%) (Fig. 2). The following reasons may be considered for this small fluctuation in the incidence of PUTIs. An explanation for PUTIs may become more common due to a combination of factors, including adequate knowledge and careful control over antibiotic use in medical procedures [8, 19, 20]. On the other hand, the improvement of medical level, preoperative cystoscopy is routinely performed. Routine cystoscopy will improve the detection rate of injury to the urinary tract [2, 21–23].

Our results showed that the higher risk of PUTIs increased with age (Fig. 3). The frequency of UTI rises with age, and in women over 65, it is around twice as high as in the general female population [24]. Medina et al. found that the cause of spinal cord dysfunction, comorbidities, age, residential status (institutionalized or not), history of antibiotic usage, and spinal cord dysfunction all play a role in the etiology of this condition in older women [24]. Women, elderly, or had comorbidities of cerebrovascular accident or chronic renal failure patients have a higher risk of developing PUTIs, according to research by Lin et al [25]. A study conducted in 2005 found that there was a statistically significant difference in the incidence of UTI diagnoses between subjects 65 years of age and older and younger subjects (16% vs. 4%, *P* < 0.05) [26]. Age is an important factor that affects a patient’s overall health, wound healing, limb mobility, and recovery rate after surgery [27]. Another reason could be that changes that last for a long time in the vaginal flora and vulvovaginal atrophy make defenses against pathogens weaker [28, 29], which makes UTIs more likely in women who have gone through menopause.

A retrospective study conducted by Sako et al. [30], showed that risk factors for low survival include male sex, older age, low bed capacity, and non-academic hospitals. The above studies were limited to small samples, and the evidence was not convincing, while the sample size of this study is large enough to be convincing. Our results suggest that there are risk factors for PUTIs: bed size of hospital (medium, large); teaching hospital; region of hospital (south, west). Teaching hospitals and large medical centers often serve as primary healthcare providers for a significant population [31]. They are equipped with advanced diagnostic facilities and specialists, making them more capable of diagnosing and treating UTIs. As a result, UTIs are more frequently detected and managed in these settings. These patients may have a higher likelihood of developing UTIs due to factors such as indwelling catheters, compromised immune systems, or underlying medical conditions. This may be related to different groups and comorbidities, family history and genetic predisposition, regional climatic factors, poor hygiene, and overuse of antibiotics [32, 33].

In line with previous studies, our results also showed that the occurrence of PUTIs in the perioperative period of TH increased hospital costs and the length of hospital stay [26, 34, 35]. Pokrzywa et al [36]. found that an increased rate of 30-d complications in elective general surgery patients with UTIs by the American College of Surgeons National Surgical Quality Improvement Program database from 2011 to 2013. However, this study only looked at the relationship between PUTIs and postoperative complications from 2011 to 2013, while our study looked at the relationship between PUTIs and postoperative complications from 2010 to 2019, and conducted an economic analysis. In 25 hospitals across China, Zhu et al [37]. looked into the prevalence, incidence, and risk factors for UTI in immobile patients. They discovered that the following factors were independent risk factors for UTI: older age, female sex, diabetes mellitus, longer hospital stays, being in a medical ward, the presence of an indwelling urethral catheter, prolonged catheter use, glucocorticoid use, and the presence of an indwelling catheter. However, this study only surveyed the population of immobile hospitalized patients.

Our findings also showed that the black population was a protective factor for PUTIs. The main considerations may be genetic variation, lifestyle habits, and past medical history, and environmental economic factors [38]. Some studies [39, 40] have suggested that genetic factors could contribute to the lower incidence of UTIs in certain racial groups, including black populations. It found variations in genes related to immune system function that could influence susceptibility to UTIs. Some research [41, 42] has indicated that black individuals in certain regions may have low economic level, less use of antibiotics or may engage in behaviors that reduce UTI risk, although this varies widely depending on location and socioeconomic status. It’s important to note that while these associations exist, they are not absolute or universally applicable. UTIs can affect individuals of all races and socioeconomic backgrounds. Factors such as personal hygiene practices, sexual activity, underlying health conditions, and antibiotic use also significantly influence UTI risk.

Our results showed that a number of comorbidities (≥ 1) was associated with UTIs (Fig. 3). Diabetes mellitus is linked to a higher risk of UTIs due to multiple factors, including chronic hyperglycemia, compromised leukocyte function, recurrent UTIs, and abnormalities in the anatomy and function of the urinary tract [18, 43]. Lin et al. [25] revealed that the incidence of high UTIs was substantially correlated with hypertension (HTN), diabetes, coronary heart disease, hyperlipidemia, chronic renal failure, cerebrovascular accidents, and depression. Bausch et al. [19] discovered that in a risk factor study of UTIs in 665 patients, worse health, higher comorbidities, concomitant immunodeficiency, and end-stage renal failure/hemodialysis were related to an increased risk of postoperative UTIs. This shows that effective interventions are needed to lower the incidence of PUTIs in people who have these traits. It has been demonstrated that active counseling with patients in these comorbidity categories lowers the occurrence of PUTIs [26]. It is worthwhile to investigate how certain therapies affect the prevalence of PUTIs. Prospective research on the actual prevalence of PUTIs at the time of admission and during hospital stays is also necessary.

Courtney et al. [36] discovered that the risk of postoperative complications and morbidity can be increased by PUTIs. Complications including congestive heart failure, wound infection, weight loss, and perioperative blood transfusion were more common in the UTIs group [36]. James et al. [44] discovered that 30 days after surgery, post-operative infections and negative outcomes were linked to UTIs. UTIs have been linked to higher rates of morbidity and death in patients after colorectal surgery. Additionally, patients who have UTIs are more likely to experience various comorbidities, including sepsis, surgical site infections, and pulmonary embolism [45]. The studies were retrospective and had small sample sizes, inadequate evidence, and an incomplete analysis of complications. One of the strengths of this study was that we used the NIS database to assess a sizable cohort of women who had TH, and we also included surgical and medical problems in the multivariate logistic regression analysis.

Our study has many limitations. One limitation of this study lies in its retrospective methodology, which inherently increases the risk of bias in both measurement and selection. While we enrolled all eligible patients between 2010 and 2019 to mitigate sample bias and reduce the impact of selection bias, it’s important to acknowledge that retrospective analyses can still introduce biases related to data collection, completeness, and accuracy. The second is that our analysis was limited to the variables that existed in the database. For example, the study does not include some need for total resection of uterine disease in the department of gynecology (cervical cancer, uterine benign disease, abnormal uterine bleeding, etc.). This may reduce the external validity of the findings; Previous studies [18] have reported that the incidence of urinary tract infection in these diseases is not very different. Another potential limitation of retrospective analysis is the underestimation of the incidence of PUTIs due to the absence of preoperative and postoperative follow-up information. There is no way to determine if patients had pre-existing UTI’s prior surgery. The NIS database only includes the patient’s baseline information, preoperative comorbidities, and postoperative complications, but does not include the patient’s uterine size, operation duration, urethral catheter indwelling time, anesthesia-related information, long-term complications, and long-term follow-up data [20]. Lastly, factors strongly associated in our analysis may be confounded by variables such as malignancy or renal failure, among others. Because these diseases are more prone to PUTIs due to the treatment of immune and immunosuppressive drugs and urinary catheter placement, prospective studies with large samples are needed in the future to reduce the influence of these confounding factors.

Despite these limitations, we believe that this study includes a sufficient sample size to test existing hypotheses, and provides an accurate representation of the incidence of PUTIs from clinical practice in academic centers, and provides some risk factors for PUTIs after TH to aid in preoperative counseling and planning for additional measures to avoid PUTIs in patients who are expected to be at higher risk of PUTIs.

## Conclusion

In conclusion, this study aimed to investigate the incidence and risk factors associated with PUTIs following TH using the NIS database. The study identified several risk factors for PUTIs during the perioperative period of TH, including (Fig S1): age ≥ 45 years, the number of comorbidities (≥ 1), hospital bed size (medium, large), teaching hospital, hospital region (south, west), preoperative comorbidities (drug abuse, alcohol abuse, hypothyroidism, lymphoma, fluid and electrolyte disorders, metastatic cancer, other neurological disorders, paralysis, peripheral vascular disorders, psychoses, pulmonary circulation disorders, renal failure, solid tumor without metastasis, valvular disease, weight loss), and complications (sepsis, acute myocardial infarction, deep vein thrombosis, gastrointestinal hemorrhage, pneumonia, stroke, wound infection, wound rupture, hemorrhage, pulmonary embolism, blood transfusion, postoperative delirium).

The findings suggest that identifying these risk factors can lead to improved preventive strategies and management of PUTIs in TH patients. Future studies may focus on exploring PUTIs following TH in specific patient populations and surgical procedures to enhance tailored interventions.

### Electronic supplementary material

Below is the link to the electronic supplementary material.

Supplementary Material 1

Supplementary Material 2

Supplementary Material 3

Supplementary Material 4

Supplementary Material 5

## Data Availability

This study is based on data provided by Nationwide Inpatient Sample (NIS) database, part of the Healthcare Cost and Utilization Project, Agency for Healthcare Research and Quality. The NIS database is a large publicly available full-payer inpatient care database in the United States and the direct web link to the database is https://www.ahrq.gov/data/hcup/index.html. Therefore, individual or grouped data cannot be shared by the authors.

## Abbreviations

PUTIs: Perioperative Urinary tract infections
TH: Total hysterectomy
UTIs: Urinary tract infections
NIS: Nationwide Inpatient Sample
LOS: Length of stay
POP: Pelvic organ prolapse
HTN: Hypertension
ICD-9: International Classification of Disease, 9th edition
ICD-10: International Classification of Disease, 10th edition
OR: Odds ratios
