# Correction: A retrospective analysis of the incidence and risk factors of perioperative urinary tract infections after total hysterectomy

**DOI:** 10.1186/s12905-024-03224-7

**Published:** 2024-06-28

**Authors:** Xianghua Cao, Yunyun Tu, Xinyao Zheng, Guizhen Xu, Qiting Wen, Pengfei Li, Chuan Chen, Qinfeng Yang, Jian Wang, Xueping Li, Fang Yu

**Affiliations:** 1 Department of Anesthesiology, Dongguan Tungwah hospital, Dongguan, China; 2 Department of Anesthesia, Longyan First Affiliated Hospital of Fujian Medical University, Longyan, Fujian 364000 China; 3 Department of Dermatology, Nanfang Hospital, Southern Medical University, Guangzhou, Guangdong 510515 China; 4 Department of Obstetrics and Gynecology, Core Facility Center, Division of Life Sciences and Medicine, The First Affiliated Hospital of USTC, University of Science and Technology of China, Hefei, Anhui 230001 China; 5 Division of Orthopaedic Surgery, Department of Orthopaedics, Nanfang Hospital, Southern Medical University, Guangzhou, Guangdong 510515 China; 6 Division of Orthopaedic Surgery, People’s Hospital of Ganzhou, No. 17 Hongqi Avenue, Zhanggong District, Ganzhou, 341000 China

**Correction: BMC Women’s Health (2024) 24:311**

10.1186/s12905-024-03153-5

Following publication of the original article [1], the order of affiliation were incorrectly given as

Author details

^1^Department of Anesthesiology, Dongguan Tungwah hospital, Dongguan, China

^2^Division of Orthopaedic Surgery, People’s Hospital of Ganzhou, No. 17 Hongqi Avenue, Zhanggong District, Ganzhou 341000, China

^3^Department of Anesthesia, Longyan First Affiliated Hospital of Fujian Medical University, Longyan, Fujian 364000, China

^4^Department of Dermatology, Nanfang Hospital, Southern Medical University, Guangzhou, Guangdong 510515, China

^5^Department of Obstetrics and Gynecology, Core Facility Center, Division of Life Sciences and Medicine, The First Affiliated Hospital of USTC, University of Science and Technology of China, Hefei, Anhui 230001, China

^6^Division of Orthopaedic Surgery, Department of Orthopaedics, Nanfang Hospital, Southern Medical University, Guangzhou, Guangdong 510515, China

but should have been

Author details

^1^Departments of Anesthesiology, Dongguan Tungwah hospital, Dongguan, China.

^2^Department of Anesthesia, Longyan First Affiliated Hospital of Fujian Medical University, Longyan, Fujian 364000, China.

^3^Department of Dermatology, Nanfang Hospital, Southern Medical University, Guangzhou, Guangdong, 510515, China.

^4^Department of Obstetrics and Gynecology, Core Facility Center, The First Affiliated Hospital of USTC, Division of Life Sciences and Medicine, University of Science and Technology of China, Hefei, Anhui 230001, China.

^5^Division of Orthopaedic Surgery, Department of Orthopaedics, Nanfang Hospital, Southern Medical University, Guangzhou, Guangdong, 510515, China.

^6^Division of Orthopaedic Surgery, People’s Hospital of Ganzhou, No. 17 Hongqi Avenue, Zhanggong District, Ganzhou, 341000, China.

The original article has been updated.

## References

[1] Cao X, Tu Y, Zheng X (2024). A retrospective analysis of the incidence and risk factors of perioperative urinary tract infections after total hysterectomy. BMC Womens Health.

