# Supplementary material for: A retrospective analysis of the incidence and risk factors of perioperative urinary tract infections after total hysterectomy
Source: BMC Womens Health. 2024 May 29;24:311. doi: 10.1186/s12905-024-03153-5 (PMC11134670; doi:10.1186/s12905-024-03153-5)
Supplement: Supplementary file 1 — Supplementary Material 1 [file 12905_2024_3153_MOESM1_ESM.docx]

**Table S1** Risk factors associated with PUTIs after TH

| **Variable** | | **Multivariate Logistic Regression** | | |
| --- | --- | --- | --- | --- |
|  |  | **OR** | **95% CI** | **P** |
| **Age** | |  |  |  |
| 18-44 | | Ref | —— | —— |
| 45-64 | | 1.20 | 1.14-1.27 | ＜0.001 |
| 65-74 | | 1.75 | 1.60-1.91 | ＜0.001 |
| ≥75 | | 2.42 | 2.19-2.67 | ＜0.001 |
| **Race** | |  |  |  |
|  | White | Ref | —— | —— |
|  | Black | 0.90 | 0.85-0.95 | ＜0.001 |
|  | Hispanic | 0.96 | 0.90-1.02 | 0.196 |
|  | Asian or Pacific Islander | 1.01 | 0.89-1.14 | 0.899 |
|  | Native American | 1.27 | 0.98-1.64 | 0.077 |
|  | Other | 0.94 | 0.83-1.06 | 0.287 |
| **Number of Comorbidity** | |  |  |  |
|  | 0 | Ref | —— | —— |
|  | 1 | 1.41 | 1.32-1.52 | ＜0.001 |
|  | 2 | 1.94 | 1.81-2.08 | ＜0.001 |
|  | ≥3 | 3.77 | 3.53-4.02 | ＜0.001 |
| **Type of insurance** | |  |  |  |
|  | Medicare | Ref | —— | —— |
|  | Medicaid | 0.96 | 0.88-1.05 | 0.355 |
|  | Private insurance | 0.72 | 0.67-0.78 | ＜0.001 |
|  | Self-pay | 1.17 | 1.04-1.32 | 0.009 |
|  | No charge | 1.09 | 0.86-1.39 | 0.459 |
|  | Other | 0.77 | 0.66-0.89 | ＜0.001 |
| **Bed size of hospital** | |  |  |  |
|  | Small | Ref | —— | —— |
|  | Medium | 1.15 | 1.07-1.24 | ＜0.001 |
|  | Large | 1.21 | 1.13-1.30 | ＜0.001 |
| **Elective admission** | | 0.32 | 0.30-0.33 | ＜0.001 |
| **Teaching hospital** | | 1.17 | 1.11-1.23 | ＜0.001 |
| **Urban hospital** | | 1.11 | 1.01-1.22 | 0.25 |
| **Region of hospital** | |  |  |  |
|  | Northeast | Ref | —— | —— |
|  | Midwest or North Central | 1.12 | 1.04-1.20 | 0.002 |
|  | South | 1.17 | 1.10-1.24 | ＜0.001 |
|  | West | 1.14 | 1.05-1.22 | ＜0.001 |

AIDS: Acquired immunodeficiency syndrome, OR: Odds ratio, CI: Confidence interval, TH: Total hysterectomy，PUTIs：Perioperative urinary tract infections
