# Supplementary material for: A retrospective analysis of the incidence and risk factors of perioperative urinary tract infections after total hysterectomy
Source: BMC Womens Health. 2024 May 29;24:311. doi: 10.1186/s12905-024-03153-5 (PMC11134670; doi:10.1186/s12905-024-03153-5)
Supplement: Supplementary file 2 — Supplementary Material 2 [file 12905_2024_3153_MOESM2_ESM.docx]

**Table S2** Relationship between PUTIs and preoperative comorbidities

| **Comorbidities** | | **Univariate Analysis** | | | **Multivariate Logistic Regression** | | | |
| --- | --- | --- | --- | --- | --- | --- | --- | --- |
|  |  | **No PUTIs** | **PUTIs** | **P** | | **OR** | **95% CI** | **P** |
| **Preoperative comorbidities** | |  |  |  | |  |  |  |
|  | Acquired immune deficiency syndrome | 593 (0.1%) | 18 (0.2%) | 0.080 | | 1.16 | 0.71-1.88 | 0.557 |
|  | Alcohol abuse | 1725 (0.4%) | 108 (1.2%) | ＜0.001 | | 1.64 | 1.33-2.03 | *＜0.001* |
|  | Deficiency anemia | 41,129 (9.4%) | 1452 (16.0%) | ＜0.001 | | 1.53 | 1.44-1.63 | *＜0.001* |
|  | Rheumatoid arthritis/collagen vascular diseases | 6059 (1.4%) | 177 (1.9%) | ＜0.001 | | 1.09 | 0.93-1.27 | 0.286 |
|  | Chronic blood loss anemia | 21,117 (4.8%) | 581 (6.4%) | ＜0.001 | | 1.34 | 1.22-1.46 | *＜0.001* |
|  | Congestive heart failure | 4,577 (1.0%) | 484 (5.3%) | ＜0.001 | | 1.55 | 1.39-1.73 | *＜0.001* |
|  | Chronic pulmonary disease | 43,803 (10.0%) | 1,278(14.1%) | ＜0.001 | | 1.10 | 1.03-1.17 | *0.003* |
|  | Coagulopathy | 6,006 (1.4%) | 468 (5.2%) | ＜0.001 | | 1.55 | 1.39-1.72 | ＜0.001 |
|  | Depression | 37,323 (8.6%) | 1,026 (11.3%) | ＜0.001 | | 1.10 | 1.03-1.18 | 0.006 |
|  | Diabetes, uncomplicated | 41,425 (9.5%) | 1,467 (16.1%) | ＜0.001 | | 1.39 | 1.31-1.48 | ＜0.001 |
|  | Diabetes, complicated | 6,151(1.4%) | 507(5.6%) | ＜0.001 | | 1.64 | 1.47-1.82 | ＜0.001 |
|  | Drug abuse | 2,907 (0.7%) | 136 (1.5%) | ＜0.001 | | 1.54 | 1.28-1.86 | ＜0.001 |
|  | Hypertension | 134,234(30.8%) | 4,257 (46.8%) | ＜0.001 | | 1.31 | 1.25-1.37 | ＜0.001 |
|  | Hypothyroidism | 41,290 (9.5%) | 1,125(12.4%) | ＜0.001 | | 1.07 | 1.00-1.14 | *＜0.001* |
|  | Liver disease | 4,566 (1.0%) | 230 (2.5%) | ＜0.001 | | 1.18 | 1.02-1.37 | 0.24 |
|  | Lymphoma | 470 (0.1%) | 31 (0.3%) | ＜0.001 | | 2.02 | 1.37-2.97 | *＜0.001* |
|  | Fluid and electrolyte disorders | 21,238 (4.9%) | 2,415 (26.6%) | ＜0.001 | | 3.78 | 3.57-4.00 | *＜0.001* |
|  | Metastatic cancer | 13,291 (3.0%) | 887 (9.8%) | ＜0.001 | | 1.67 | 1.52-1.80 | *＜0.001* |
|  | Other neurological disorders | 6,969 (1.6%) | 467 (5.1%) | ＜0.001 | | 1.86 | 1.67-2.07 | *＜0.001* |
|  | Obesity | 74,793 (17.1%) | 2,114 (23.3%) | ＜0.001 | | 1.01 | 1.02-1.14 | *0.006* |
|  | Paralysis | 1,062 (0.2%) | 162 (1.8%) | ＜0.001 | | 3.83 | 3.19-4.60 | *＜0.001* |
|  | Peripheral vascular disorders | 2,748 (0.6%) | 195 (2.1%) | ＜0.001 | | 1.38 | 1.18-1.62 | *＜0.001* |
|  | Psychoses | 7,905 (1.8%) | 294(3.2%) | ＜0.001 | | 1.35 | 1.19-1.52 | *＜0.001* |
|  | Pulmonary circulation disorders | 2,312 (0.5%) | 295 (3.2%) | ＜0.001 | | 2.01 | 1.80-2.37 | *＜0.001* |
|  | Renal failure | 6,241 (1.4%) | 611 (6.7%) | ＜0.001 | | 1.52 | 1.37-1.68 | *＜0.001* |
|  | Solid tumor without metastasis | 26,724 (6.1%) | 1,177 (13.0%) | ＜0.001 | | 1.26 | 1.17-1.36 | *＜0.001* |
|  | Peptic ulcer disease excluding bleeding | 219(0.1%) | 9(0.1%) | 0.042 | | 0.72 | 0.35-1.46 | *0.362* |
|  | Valvular disease | 6,408 (1.5%) | 301 (3.3%) | ＜0.001 | | 1.19 | 1.05-1.36 | *＜0.001* |
|  | Weight loss | 4,124 (0.9%) | 786(8.6%) | ＜0.001 | | 3.06 | 2.79-3.35 | *＜0.001* |

OR: Odds ratio, CI: Confidence interval, PUTIs：Perioperative urinary tract infections, No PUTIs: No perioperative urinary tract infections
