# Supplementary material for: A retrospective analysis of the incidence and risk factors of perioperative urinary tract infections after total hysterectomy
Source: BMC Womens Health. 2024 May 29;24:311. doi: 10.1186/s12905-024-03153-5 (PMC11134670; doi:10.1186/s12905-024-03153-5)
Supplement: Supplementary file 3 — Supplementary Material 3 [file 12905_2024_3153_MOESM3_ESM.docx]

**Table S3** Relationship between PUTIs and postoperative complications

| **Complications** | | **Univariate Analysis** | | | **Multivariate Logistic Regression** | | |
| --- | --- | --- | --- | --- | --- | --- | --- |
|  |  | **No PUTIs** | **PUTIs** | **P** | **OR** | **95% CI** | **P** |
| **Medical complications** | |  |  |  |  |  |  |
|  | Sepsis | 2,155 (0.5%) | 702 (7.7%) | ＜0.001 | 6.19 | 5.57-6.88 | *＜0.001* |
|  | Acute myocardial infarction | 1,407 (0.3%) | 139(1.5%) | ＜0.001 | 1.89 | 1.54-2.31 | *＜0.001* |
|  | Deep vein thrombosis | 1,605 (0.4%) | 267(2.9%) | ＜0.001 | 2.63 | 2.24-3.09 | *＜0.001* |
|  | Gastrointestinal hemorrhage | 373 (0.1%) | 85(0.9%) | ＜0.001 | 2.79 | 2.11-3.70 | *＜0.001* |
|  | Cardiac arrest | 295(0.1%) | 26 (0.3%) | 0.049 | 0.55 | 0.35-0.88 | 0.013 |
|  | Shock | 684 (0.2%) | 86(0.9%) | ＜0.001 | 1.48 | 1.14-1.92 | 0.003 |
|  | Pneumonia | 2,593 (0.6%) | 447(4.9%) | ＜0.001 | 2.80 | 2.48-3.18 | *＜0.001* |
|  | Stroke | 587 (0.1%) | 81(0.9%) | ＜0.001 | 3.62 | 2.79-4.70 | *＜0.001* |
| **Surgical complications** | |  |  |  |  |  |  |
|  | Wound infection | 1,705 (0.4%) | 336 (3.7%) | ＜0.001 | 3.04 | 2.63-3.51 | *＜0.001* |
|  | Wound rupture | 916 (0.2%) | 162(1.8%) | ＜0.001 | 2.37 | 1.93-2.91 | *＜0.001* |
|  | Hemorrhage | 5,866(1.3%) | 394(4.3%) | ＜0.001 | 1.55 | 1.38-1.74 | *＜0.001* |
|  | Pulmonary embolism | 1,460 (0.3%) | 230(2.5%) | ＜0.001 | 2.38 | 2.00-2.82 | *＜0.001* |
|  | Blood transfusion | 32,500 (7.4%) | 2,041 (22.5%) | ＜0.001 | 2.65 | 2.51-2.80 | *＜0.001* |
|  | Postoperative delirium | 792(0.2%) | 154(1.7%) | ＜0.001 | 4.17 | 3.41-5.09 | *＜0.001* |

OR: Odds ratio, CI: Confidence interval, PUTIs：Perioperative urinary tract infections, No PUTIs: No perioperative urinary tract infections
