# Supplementary figures and images for: A retrospective analysis of the incidence and risk factors of perioperative urinary tract infections after total hysterectomy
Source: BMC Womens Health. 2024 May 29;24:311. doi: 10.1186/s12905-024-03153-5 (PMC11134670; doi:10.1186/s12905-024-03153-5)

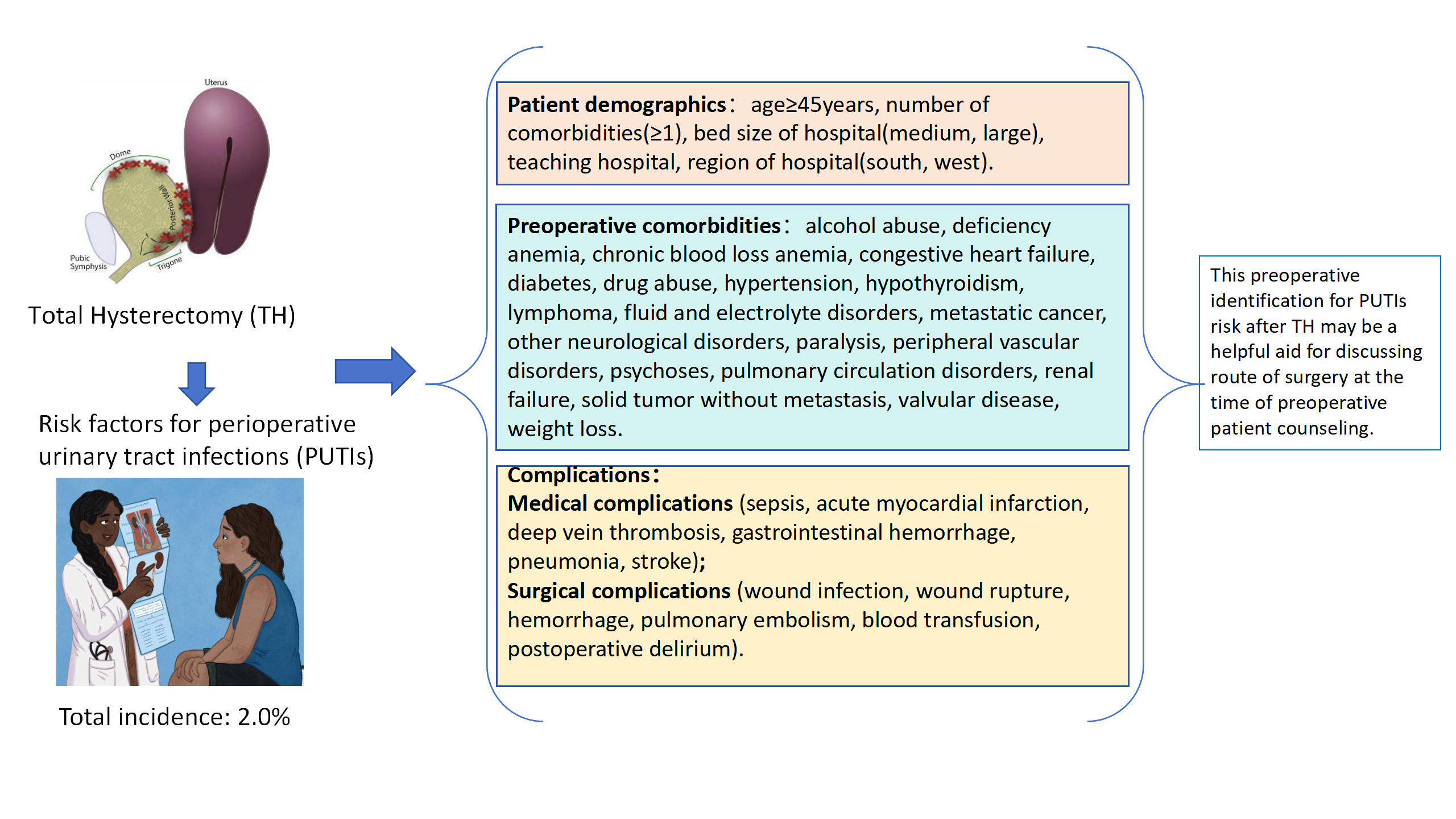

Supplement: Supplementary file 4 — Supplementary Material 4 [file 12905_2024_3153_MOESM4_ESM.tif]
