# Supplementary material for: A retrospective analysis of the incidence and risk factors of perioperative urinary tract infections after total hysterectomy
Source: BMC Womens Health. 2024 May 29;24:311. doi: 10.1186/s12905-024-03153-5 (PMC11134670; doi:10.1186/s12905-024-03153-5)
Supplement: Supplementary file 5 — Supplementary Material 5 [file 12905_2024_3153_MOESM5_ESM.docx]

**Figure S1.** Overview of risk factors associated with PUTIs after TH

**Table S1**. Risk factors associated with PUTIs after TH.

**Table S2**. Relationship between PUTIs and preoperative comorbidities.

**Table S3**. Relationship between PUTIs and postoperative complications.
